# Joint EANM/SIOPE/RAPNO practice guidelines/SNMMI procedure standards for imaging of paediatric gliomas using PET with radiolabelled amino acids and [^18^F]FDG: version 1.0

**DOI:** 10.1007/s00259-022-05817-6

**Published:** 2022-05-10

**Authors:** Arnoldo Piccardo, Nathalie L. Albert, Lise Borgwardt, Frederic H. Fahey, Darren Hargrave, Norbert Galldiks, Nina Jehanno, Lars Kurch, Ian Law, Ruth Lim, Egesta Lopci, Lisbeth Marner, Giovanni Morana, Tina Young Poussaint, Victor J. Seghers, Barry L. Shulkin, Katherine E. Warren, Tatjana Traub-Weidinger, Pietro Zucchetta

**Affiliations:** 1 Department of Nuclear Medicine, E.O. “Ospedali Galliera”, Genoa, Italy; 2 Department of Nuclear Medicine, University Hospital of LMU Munich, Munich, Germany; 3 Department of Clinical Physiology, Nuclear Medicine and PET, Rigshospitalet, University of Copenhagen, Copenhagen, Denmark; 4 Department of Radiology, Boston Children’s Hospital, Harvard Medical School, Boston, MA USA; 5 Department of Paediatric Oncology, Great Ormond Street Hospital NHS Trust, London, UK; 6 Department of Neurology, Faculty of Medicine and University Hospital Cologne, University of Cologne, Cologne, Germany; 7 Institute of Neuroscience and Medicine (INM-3), Research Center Juelich, Juelich, Germany; 8 Department of Nuclear Medicine, Institut Curie Paris, Paris, France; 9 Department of Nuclear Medicine, University Hospital Leipzig, Leipzig, Germany; 10 Department of Radiology, Massachusetts General Hospital, Boston, MA USA; 11 Nuclear Medicine Unit, IRCCS-Humanitas Research Hospital, Via Manzoni 56, 20089 Rozzano, Milano Italy; 12 Department of Clinical Physiology and Nuclear Medicine, Copenhagen University Hospital Bispebjerg, Copenhagen, Denmark; 13 Department of Neurosciences, University of Turin, Turin, Italy; 14 Singleton Department of Pediatric Radiology, Texas Children’s Hospital, Houston, TX USA; 15 Department of Radiology, Baylor College of Medicine, Houston, TX USA; 16 Nuclear Medicine Department of Diagnostic Imaging St. Jude Children’s Research Hospital, University of Tennessee Health Science Center, Memphis, TN USA; 17 Department of Pediatric Oncology, Dana Farber Cancer Institute, Boston, MA USA; 18 Division of Nuclear Medicine, Department of Biomedical Imaging and Image-Guided Therapy, Medical University of Vienna, Vienna, Austria; 19 Nuclear Medicine Unit, Department of Medicine - DIMED, University Hospital of Padova, Padua, Italy

**Keywords:** PET-CT, Paediatric PET, Paediatric oncology, Paediatric brain imaging, FDG, FET, MET, DOPA, Gliomas

## Abstract

Positron emission tomography (PET) has been widely used in paediatric oncology. 2-Deoxy-2-[^18^F]fluoro-D-glucose ([^18^F]FDG) is the most commonly used radiopharmaceutical for PET imaging. For oncological brain imaging, different amino acid PET radiopharmaceuticals have been introduced in the last years. The purpose of this document is to provide imaging specialists and clinicians guidelines for indication, acquisition, and interpretation of [^18^F]FDG and radiolabelled amino acid PET in paediatric patients affected by brain gliomas. There is no high level of evidence for all recommendations suggested in this paper. These recommendations represent instead the consensus opinion of experienced leaders in the field. Further studies are needed to reach evidence-based recommendations for the applications of [^18^F]FDG and radiolabelled amino acid PET in paediatric neuro-oncology. These recommendations are not intended to be a substitute for national and international legal or regulatory provisions and should be considered in the context of good practice in nuclear medicine. The present guidelines/standards were developed collaboratively by the EANM and SNMMI with the European Society for Paediatric Oncology (SIOPE) Brain Tumour Group and the Response Assessment in Paediatric Neuro-Oncology (RAPNO) working group. They summarize also the views of the Neuroimaging and Oncology and Theranostics Committees of the EANM and reflect recommendations for which the EANM and other societies cannot be held responsible.

## Preface

The European Association of Nuclear Medicine (EANM) is a professional non-profit medical association founded in 1985 to facilitate worldwide communication among individuals pursuing clinical and academic excellence in nuclear medicine. The Society of Nuclear Medicine and Molecular Imaging (SNMMI) is an international scientific and professional organization founded in 1954 to promote science, technology, and practical application of nuclear medicine. SNMMI and EANM members are physicians, technologists, physicists, and scientists specialized in the research and clinical practice of nuclear medicine.

The SNMMI and EANM will periodically put forth new standards/guidelines for nuclear medicine practice to help advance the science of nuclear medicine and improve service to patients.

Existing standards/guidelines will be reviewed for revision or renewal, as appropriate, on their fifth anniversary or sooner, if indicated. Each standard/guideline, representing a policy statement by the SNMMI/EANM, has undergone a thorough consensus process, entailing extensive review. The SNMMI and EANM recognize that the safe and effective use of diagnostic nuclear medicine imaging requires particular training and skills, as described in each document. Reproduction or modification of the published practice guideline by those entities not providing these services is not authorized.

These standards/guidelines are educational tools designed to assist practitioners in providing appropriate and effective nuclear medicine care for patients. These guidelines are consensus documents and are not inflexible rules or requirements of practice. They are not intended, nor should they be used, to establish a legal standard of care. For these reasons and those set forth below, the SNMMI and the EANM cautions against the use of these standards/guidelines in litigation in which the clinical decisions of a practitioner are called into question.

The ultimate judgement regarding the propriety of any specific procedure or course of action must be made by the physician or medical physicist in light of all the circumstances presented. Thus, there is no implication that an action differing from what is laid out in the standards/guidelines, standing alone, is below standard of care. To the contrary, a conscientious practitioner may responsibly adopt a course of action different from that set forth in the standards/guidelines when, in the reasonable judgement of the practitioner, such course of action is indicated by the condition of the patient, limitations of available resources, or advances in knowledge or technology subsequent to publication of the standards/guidelines.

The practice of medicine involves not only the science, but also the art of dealing with the prevention, diagnosis, alleviation, and treatment of disease. The variety and complexity of human conditions make it impossible for general guidelines to consistently allow for an accurate diagnosis to be reached or a particular treatment response to be predicted. Therefore, it should be recognized that adherence to these standards/guidelines will not ensure a successful outcome. All that should be expected is that the practitioners follow a reasonable course of action, based on their level of training, the current knowledge, the available resources, and the needs/context of the particular patient to deliver effective and safe medical care. The sole purpose of these guidelines is to assist practitioners in achieving this objective.

Positron emission tomography (PET) has been widely used in paediatric oncology. 2-Deoxy-2-[^18^F]fluoro-D-glucose ([^18^F]FDG) is the most commonly used radiopharmaceutical for PET imaging. For oncological brain imaging different amino acid PET radiopharmaceuticals have been introduced in the last years. The purpose of this document is to provide imaging specialists and clinicians guidelines for indication, acquisition, and interpretation of [^18^F]FDG and radiolabelled amino acid PET in paediatric patients affected by brain gliomas. There is no high level of evidence for all recommendations suggested in this paper. These recommendations represent instead the consensus opinion of experienced leaders in the field. Further studies are needed to reach evidence-based recommendations for the applications of [^18^F]FDG and radiolabelled amino acid PET in paediatric neuro-oncology. These recommendations are not intended to be a substitute for national and international legal or regulatory provisions and should be considered in the context of good practice in nuclear medicine.

The present guidelines/standards were developed collaboratively by the EANM and SNMMI with the European Society for Paediatric Oncology (SIOPE) Brain Tumour Group and the Response Assessment in Paediatric Neuro-Oncology (RAPNO) working group. They summarize also the views of the Neuroimaging and Oncology and Theranostics Committees of the EANM and reflect recommendations for which the EANM and other societies cannot be held responsible.

## Introduction

Paediatric brain tumours include a heterogeneous variety of malignancies, which all present specific biological, prognostic, and treatment-related features. Tumours arising within the central nervous system (CNS) are, from the epidemiological point of view, the most frequent solid paediatric malignancy and the second most common after leukaemia. Indeed, they cause up to 27% of all malignancies in children aged 14 and younger and up to 10% of those occurring in adolescents between 15 and 19 years of age. Finally, CNS neoplasm are the main cause of paediatric cancer-related death [1, 2].

Paediatric brain gliomas are the most frequent central nervous system tumours in childhood and comprise a heterogeneous collection of neoplasms (including astrocytic and mixed neuronal-glial tumours), which varies from low-grade to highly aggressive malignancies, and can present in a diffusely infiltrating or a more circumscribed growth pattern [3–7]. When compared with adult gliomas, paediatric gliomas show divergent mechanisms of tumorigenesis, distinct molecular genetic alterations, and different clinical behaviour. In particular, low-grade paediatric gliomas evolve to their high-grade counterparts infrequently [8]. Therefore, paediatric gliomas are considered biologically distinct entities [3–5]. All these aspects were recently recognized in the 2021 fifth edition of the World Health Organization (WHO) Classification of Tumours of the CNS (CNS5) where diffuse adult-type gliomas were classified separately from those of the paediatric population (“paediatric-type” gliomas) [9].

Paediatric low-grade gliomas (pLGG) are defined as grade 1 or 2 malignancies according to the WHO classification. These tumours include many different histological subtypes that can manifest throughout the CNS. The most common entity is pilocytic astrocytoma (a circumscribed astrocytic glioma), whereas paediatric-type diffuse low-grade gliomas and glioneuronal and neuronal tumours compose a notable minority. The most frequent genomic alterations in pLGGs are related to an activation of the mitogen-activated protein kinase (MAPK) pathway [5, 10]. This alteration can occur in presence of gene rearrangement, due to a tandem duplication at 7q34 determining a fusion between KIAA1549 and BRAF (KIAA1549-BRAF fusion) or by point mutation at the codon 600, which results in an amino acid substitution from valine (V) to glutamic acid (E) (BRAF V600E mutation) [10, 11]. KIAA1549-BRAF fusion is present in up to 66% of pilocytic astrocytomas and in 15% of all other low-grade gliomas, and it is recognized as a factor associated with a better prognosis [10, 12]. In addition, a further gene possibly involved in tumorigenesis is the one encoding the isocitrate dehydrogenase (IDH) enzyme, which catalyse the oxidative decarboxylation of isocitrate and therefore has a pivotal role in cell energy production. This gene has been indeed found to be mutated in gliomas [13]. When compared with adult lower-grade gliomas, IDH mutations are less frequent in the paediatric population particularly in younger children, and malignant progression is extremely rare in paediatric IDH wild-type LGGs [3].

Paediatric high-grade gliomas (pHGG), on the other hand, are one of the main causes of cancer-related death in children. These include various WHO grade 3 and 4 entities (some of them newly recognized in the WHO CNS5 under the group of paediatric-type diffuse high-grade gliomas), as well as diffuse midline glioma, H3K27-altered first introduced in the 2016 WHO classification [6]. The detection of H3K27 alteration, independent of the histological appearance (which could even be that of a low grade diffusely infiltrating lesion), constitutes classification as a WHO grade 4 [6]. Diffuse midline gliomas (DMG) can arise in any of the central nervous system mid-line structures (e.g. the brain stem, the thalamus, and the spinal cord). The H3K27 alteration is observed in up to 85% of diffuse intrinsic pontine gliomas (DIPG), which are aggressive malignant brainstem DMG for which the median survival is less than 1 year [14]. Of note, DIPG diagnosis can still be made based on clinical and imaging features alone, without tissue sampling. From the clinical point of view, these tumours manifest with a characteristic triad: multiple cranial neuropathies, long tract signs (hyperreflexia, clonus, increased tone, Babinski reflex), and ataxia. Classic features on MRI are a T1 hypointense and T2 hyperintense diffusely infiltrating lesion arising from and involving ≥ 50% of the pons [14].

Despite similar histological characteristics, pHGGs have different molecular features when compared with adult gliomas, both in terms of mutation pattern and in the prognostic implication of those mutations. In particular, approximately 40% of pHGGs are associated with tumour suppressor gene TP53 mutations [15, 16]. Compared with adults, pHGGs are less likely to bear epidermal growth factor receptor (EGFR) gene amplification and less likely to display mutations in the tumour suppressor PTEN [16, 17]. Up to one-third of hemispheric pHGGs carry mutations at position 34 (G34R/V) in H3F3A [3]. Conversely, hotspot mutations in IDH1/2 are rare in older adolescent and represent the lower age spectrum of adult gliomas [3, 18].

It is worth noting that familial syndrome might increase the occurrence of CNS paediatric tumours: in fact, 8–19% of these neoplasms is found in patients with genetic predisposition; this figure is significantly lower in adults [19]. These conditions include tuberous sclerosis, neurofibromatosis, Li-Fraumeni syndrome, rhabdoid tumour predisposition syndrome, von Hippel-Lindau disease, naevoid basal cell carcinoma syndrome, and Turcot’s syndrome [20].

Clinical management of paediatric brain gliomas is challenging and requires a multidisciplinary approach in order to devise the best diagnostic and therapeutic strategies in each individual patient. Surgery represents the treatment of choice and can have the most relevant impact on patients’ prognosis. If complete surgical resection is not feasible, biopsy or “debulking” can be considered, and adjuvant therapy with radiotherapy, chemotherapy, or a combination of both may be used. However, for pHGGs, due to possible persistence of microscopic disease, adjuvant therapy is proposed and performed even in case of complete tumour removal. In conclusion, the principal therapeutic approach in paediatric gliomas is represented by surgery in association with chemo- and/or radiotherapy [21].

Sensitive and effective non-invasive imaging procedures are especially needed in this “era” of new surgical techniques, radiotherapy planning, and novel systemic treatment. Although conventional MRI is the pivotal imaging procedure in paediatric brain gliomas to determine tumour location, presence of oedema, presence of intratumoural necrosis, cyst formation, haemorrhage, vascularization, mass effect, and contrast enhancement, it has some limitations in distinguishing tumour from tumour mimics and in defining tumour type and grade. Moreover, it does not always allow for precise delineation of tumour margins, distinguish cells in the tumour microenvironment, or inform about the metabolism or state of tumour cells.

Indeed, after treatment, the differentiation between true tumour remnants and treatment-related changes (e.g. “pseudoprogression” or “pseudoresponse”) can be very difficult, specifically when an early response assessment is performed. Pseudoprogression occurs in approximately 21–44% of DIPG [22] and 7–12% of pHGG cases [23]; it is a subacute treatment-related reaction (occurring most frequently within the first 3 months after chemoradiotherapy) characterized by a “transient increase in tumour size due to an increase in blood–brain barrier permeability caused by treatment, resulting in increased oedema, or contrast enhancement, or both” [23].

Pseudoresponse is a different MRI phenomenon characterized by “radiographical improvement, with overall decreased oedema, or contrast enhancement secondary to the normalisation effect of antiangiogenic agents on the permeability of leaky endothelium, or both, without a change in survival outcome” [23]. Of note, differently from adult HGG, pseudoresponse is uncommon in pHGG [23].

In this setting, although response standard criteria for paediatric gliomas exist [22–24], the diffusely infiltrative pattern of pHGGs, often presenting undefined margins and inhomogeneous contrast enhancement, can complicate tumour measurement and response assessment. In this context, MRI contrast enhancement for the estimation of tumour size or growth has limited use. In fact, this parameter reflects the increased permeability of the disrupted blood-tumour barrier to the contrast medium rather than tumour-specific vascularization.

In addition, contrast enhancement can be altered by therapies that influence tumour vascular permeability, such as corticosteroid, antiangiogenic [25], or immunotherapy agents [26]. Finally, the real biological activity of gliomas on the first diagnosis is often not correctly estimated by MRI. This limits the usefulness of this imaging procedure in clinical decision-making [27].

Multiparametric MRI procedures can overcome some of these constraints being able to reveal additional hemodynamic and metabolic information of brain tumours. The lack of standardization, reproducibility, and comparability of data has reduced their clinical applications. In this context, imaging biomarkers able to provide reliable parameters of tumour biological activity are required to tailor the clinical management of paediatric patients.

In the last two decades, PET imaging has been increasingly used in the paediatric population affected by brain neoplasms, particularly in those suffering from gliomas. Different biological aspects can be studied using different radiopharmaceuticals, with increasing evidence supporting the role of amino acid PET radiopharmaceuticals for use in glioma imaging [28, 29]. PET imaging of brain tumours with amino acid analogues (namely: L amino acid transport comprising [^11^C-methyl]-methionine ([^11^C]MET), O-(2-[18F]fluoroethyl)-L-tyrosine ([^18^F]FET) and 3,4-dihydroxy-6-[^18^F]fluoro-L-phenylalanine ([^18^F]DOPA)) has shown clear advantages over [^18^F]FDG because of the better contrast between tumour and background uptake. Furthermore, considering that the uptake amino acid radiopharmaceutical does not depend on blood–brain barrier status, but rather on the expression of the amino acid transport system, these radiopharmaceuticals can be taken up in both contrast enhancing and non-enhancing tumour lesions [30, 31].

The present practical guidelines/procedure standards highlight, for the first time, the technical aspects of PET image acquisition with [^18^F]FDG and amino acid PET radiopharmaceuticals in paediatric gliomas imaging.

## Aim

These guidelines have been prepared to support nuclear medicine practitioners to optimize paediatric glioma imaging. These recommendations aim to assist the execution, the correct interpretation and reporting of PET results using [^18^F]FDG, and amino acid radiopharmaceuticals. Given the paucity of data available, no other PET radiopharmaceuticals have been included in these guidelines.

## Definitions

1. PET systems are able to provide static and dynamic images of radionuclide distribution within the patients by detecting coincidence 511 keV photon pairs. Images are produced by a reconstruction process using the coincidence pair data derived from positrons’ annihilation**.**
2. Hybrid imaging is performed with PET/CT or PET/MRI systems to allow for correlative anatomic information and additional tissue characterization.
3. The CT component of a PET/CT is performed with adjustable radiological parameters (i.e. mAs, kVp, and pitch, with or without contrast) to address specific clinical questions. However, a low-dose CT is sufficient for attenuation correction and anatomic localization.
4. Post procedural PET/MRI fusion refers to the display of co-registered, separately acquired MRI and PET images.

The radiopharmaceuticals considered in this guideline are 2-deoxy-2-[^18^F]fluoro-D-glucose ([^18^F]FDG), L-[methyl-^11^C]methionine ([^11^C]MET), O-(2-[^18^F]fluoroethyl)-L-tyrosine ([^18^F]FET), and 3,4-dihydroxy-6-[^18^F]fluoro-L-phenylalanine ([^18^F]DOPA).

## Clinical indications

At the time of diagnosis

1. Differential diagnosis between neoplastic and non-neoplastic lesions
2. Glioma grading/DIPG biological behaviour
3. Identification of the optimal biopsy site (i.e. area of highest radiopharmaceutical uptake)
4. Delineation of glioma extent before surgery and radiation therapy
5. Prognostication

At restaging and follow-up

1. Differentiation of glioma recurrence from treatment-induced changes (e.g. pseudo-progression, radio-necrosis, pseudo-response)
2. Response assessment during and after radiotherapy and/or chemotherapy
3. Detection of high-grade malignant transformation of diffuse low-grade gliomas
4. Detection of residual tumour after surgery and delineation of vital glioma tissue for planning of re-treatment (e.g. re-resection, re-irradiation)

In all these indications, amino acid PET radiopharmaceuticals play a more pertinent role as compared to [^18^F]FDG due to their low physiological brain uptake and their specific uptake by tumour cells.

## Qualification and responsibilities of personnel

### Physicians

All clinicians and personnel involved in performing and interpreting PET imaging should be qualified in accordance to the applicable regional laws. Responsibilities afforded to various individuals involved in the performance and interpretation of these scans should be documented in standard operating procedures. The scan should be supervised and interpreted by a nuclear medicine physician or diagnostic radiologist with specific training in nuclear medicine. Please also see the Society of Nuclear Medicine Procedure Guidelines for General Imaging [32].

### Technologists

PET/CT or PET/MRI scans should be performed by a qualified registered/certified nuclear medicine technologist and advisably by technologists with specific training in handling children. Please refer to the following documents for further details: Performance Responsibility and Guidelines for Nuclear Medicine Technologists 3.1 and the EANM Benchmark Document Nuclear Medicine Technologists’ Competencies [33, 34]. According to location of practice, additional qualifications may be requested for technologists to use the CT and MR component of the scanner.

### Medical physicists

PET/CT systems should comply with the international standard of quality, including dosimetry and radiation protection procedure to limit the irradiation exposure of patients and healthcare personnel. This is of particular importance in the paediatric context.

A medical physicist should therefore optimize PET/CT scan protocols, ensuring that the established standards are met. A medical physicist can assist physicians to adhere to and maintain good practice, by monitoring and optimizing radiation dose and developing algorithms to reduce the radiation exposure of the CT component. Please consult the manufacturer manuals and the following documents for further details: American College of Radiology, ACR-AAPM technical standard for medical physics performance monitoring of PET/CT imaging equipment, and the European guidelines on medical physics expert [35, 36]), Standards Publication Performance: Measurements of Positron Emission Tomographs (PET), EANM 2010, IAEA 2009 [37].

### PET/MRI

A medical physicist should supervise the safety measures regarding access to the magnetic room, as prescribed by local regulations.

Quality assurance for the PET/MRI scanner includes the usual procedures for the PET component, as for PET/CT systems (see above), considering the peculiarities related to the attenuation correction of the phantoms. Details on the topic are provided by recent studies and consensus statement [38–40]. The American College of Radiology MRI quality manual [41], the American association of Physicists in Medicine Report NO. 100 [42], offers guidance on MRI quality assurance. They should be integrated with the manufacturer’s recommendations and the local prescriptions.

The high sensitivity of PET/MRI scanners allows a reduction of the injected activity or of the acquisition time without compromising PET image quality [43, 44], depending on the clinical situation (degree of cooperation, sedation duration, etc.)

## Procedure/examination

[^18^F]FDG and amino acid PET radiopharmaceuticals’ specific procedures are defined and described in previous guidelines in paediatrics and adults, respectively [28, 45]. In this document, only the paediatric-related recommendations that are new or specific in the context of gliomas are discussed.

### Requirements

A strict collaboration among the nuclear medicine physician, paediatric radiologist, paediatric neuroradiologist, technologist, and the referring physician is required to obtain appropriate and useful images. The clinical indication, the histopathologic results (if available), any history of previous studies (e.g. MRI), timing of previous interventions/therapies (biopsy, surgery, chemotherapy, and radiotherapy), history of recent infections or inflammation, medications (e.g. corticosteroid), and whether sedation or analgesia is required are aspects which should be clarified before performing the study.

## Patient preparation and precautions

Imaging of the paediatric patient requires a proactive approach to reduce stress and discomfort to the minimum. Detailed explanation of the procedure, parents’ involvement, distracting techniques (video, lighting, wall decorations, dedicated waiting spaces, etc.), and assistance from Child Life Specialists are useful aids. Specifically, patient preparation and precautions should be always considered as follows:

1. The patients should be able to lie down and maintain the position for at least 15–20 min (30–40 min, should be considered if a dynamic study is scheduled).
2. The procedure of the study should be explained and adapted to the patients’ age. Written informed consent will be provided, when required, to the patients and/or to their parents/caregivers.
3. The patients should fast for at least 4 h. For [^18^F]FDG PET, serum glucose levels should be measured before the injection due to the potential implication on biodistribution.
4. Body height and weight should be documented for calculation of optimal dosing.
5. If the patient does not require sedation or general anaesthesia, drinking water before and after the injection is encouraged to increase the radiopharmaceutical elimination and reduce patient’s radiation exposure. Although anaesthesia should be reserved to very young or non-compliant children, it could be necessary in some conditions (e.g. long dynamic acquisition). In this case, a “nothing by mouth” preparation for 6–8 h is required, and anaesthesia should be administered immediately before the image acquisition and after the radiopharmaceutical injection (at least 30 min after for [^18^F]FDG).
6. When sedation is requested for amino acid radiopharmaceuticals imaging, it should start about 20–60 min before the examination [28]. In case of [^18^F]FDG imaging, sedation should start as late as possible after [^18^F]FDG administration, ideally at least 30 min after [^18^F]FDG injection but prior to imaging [28].
7. To reduce stress to the patients and minimize irradiation of the personnel, an optimization of radiopharmaceutical injection should be achieved by obtaining a peripheral intravenous access in advance.
8. A pregnancy test for female patients who have reached puberty should be considered, especially in the case of recent brain lesion or glioma diagnosis.
9. The patients should sit in a comfortable chair or lie down on an appropriate bed in a warm and quiet room. Particularly for [^18^F]FDG PET, patients should be instructed not to move, play or talk, and, whenever possible, to keep their eyes closed during the injection and the uptake phase [46].
10. Carbidopa premedication (2 mg/kg) 60 min before [^18^F]DOPA injection, which inhibits aromatic amino acid decarboxylase in extracerebral tissues, can increase [^18^F]DOPA brain availability, thus improving the quality of the PET images, and reduce the radiation exposure of the urinary tracts [47, 48]. However, this is an off-label indication in paediatrics, and its diagnostic benefit in neuro-oncological PET studies has not been proven. Moreover, possible drug interactions should be considered. For these reasons, its use should be considered only in selected cases when low-quality PET images are expected.
11. When choosing the protocol for the CT component, the ALARA (As Low As Reasonably Achievable) principle for reducing radiation exposure should be followed. Considering that a recent MRI should be always available to correctly interpret the PET findings, a non-diagnostic and low-dose CT without contrast should be recommended.
12. In the case of a PET/MRI study, along minimal requirements related to the PET component, paediatric patients necessitate an additional screening checklist for relevant contraindication to MRI including metallic implants and claustrophobia [49]. Moreover, given the longer acquisition time generally required for PET/MRI studies, a preliminary assessment for patient sedation or general anaesthesia must be performed. Metal (e.g. zippers, buttons, hairpins) must be removed from patients before entering the scanner room. Subjects with programmable cerebrospinal fluid shunt valves require prompt setting assessment and/or readjustment after MRI to correct changes in opening pressure induced by magnetic fields [50].

### Specific indication and performance of [^18^F]FDG PET

While also utilized for detecting seizure foci in paediatric epilepsy, [^18^F]FDG was the first PET radiopharmaceutical to be used in evaluating children with brain tumours. The rationale behind the use of [^18^F]FDG for paediatric brain tumour imaging is based on the well-known increased glucose consumption in malignant gliomas. Indeed, given the increased energy requirement of the proliferating cells, the glucose analogue is taken up by the neoplastic clone trough cell membrane glucose transporters [51]. On the other hand, the physiologic high glucose uptake in normal cerebral parenchyma results in a low tumour-to-background contrast ratio, which makes it difficult to differentiate intracerebral malignancies from normal tissue or non-tumorous lesions. This is especially true for low-grade tumours, in which [^18^F]FDG uptake is often similar to that of normal white matter [51, 52]. Consequently, the principal clinical application over the years has been the evaluation of the biological behaviour of CNS paediatric tumour. In particular, a glioma characterized by an extensively increased [^18^F]FDG uptake is often associated with a more malignant and aggressive behaviour [51]. This was observed in patients affected by typical paediatric tumours of the posterior fossa (e.g. medulloblastoma)[47], as well as for brain stem gliomas, including DIPG [47, 53]). [^18^F]FDG can play a role even in low-grade gliomas (e.g. low-grade astrocytoma), in which higher uptake signals refer to a higher probability of disease progression [54]. However, no conclusive threshold for [^18^F]FDG uptake able to identify patients at higher risk of progression has been defined. Nevertheless, a hotspot/brain index (i.e. 2 + 2 *(ROItumour-ROIwhite matter)/ (ROIgray matter-ROIwhite matter)) of 1.83 has been proposed as the best cut-off to differentiate low from high-grade tumours [51].

Different [^18^F]FDG uptake patterns have been reported in paediatric brain lesions, and attempts have been made to associate the uptake intensity and uniformity with histopathology. However, the pure intrinsic diagnostic reliability of [^18^F]FDG PET/CT is suboptimal. In fact, high values of [^18^F]FDG intensity and uniformity may be found not only in aggressive diseases as glioblastomas (GBM) and medulloblastoma, but also in low-grade lesions like pilocytic astrocytomas [55].

[^18^F]FDG PET has also been used in children to define targets for biopsy after proper co-registration with MRI. This point is of particular importance in paediatric patients affected by unresectable brain tumours with infiltrative pattern, where the biopsy cannot be guided by the area of contrast enhancement or the area with the highest signal on fluid attenuation inversion recovery (FLAIR) sequences [56]. Indeed, [^18^F]FDG PET may reveal tumour heterogeneity, thus allowing the identification of the anaplastic tissue, often corresponding to the area with the highest uptake amenable for biopsy. This approach can consequently reduce the frequency of non-contributory sampling [56].

However, regarding this issue, not concordant results have been reported, and recent evidence indicated that paediatric brain tumours were metabolically heterogeneous on [^18^F]FDG PET and magnetic resonance spectroscopic imaging (MRSI). [^18^F]FDG PET and MSRI tend to identify slightly different area of tumour activity, and therefore the agreement in the estimation of tumour metabolic activity between the two techniques is limited [57]. Particularly, active tumour metabolism was observed more frequently using MRSI compared with [^18^F]FDG PET.

### Specific indication and performance of amino acid PET radiopharmaceuticals

#### [^11^C]MET

L-[methyl-11C]Methionine ([^11^C]MET) represents the oldest amino acid PET radiopharmaceutical introduced in clinical practice to study paediatric brain tumours [58]. It is incorporated in the majority of brain gliomas by means of an active amino acid transport. Its uptake mechanism is related to cell proliferation, Ki-67 expression, number of viable tumour cells, and micro-vessel density. For these reasons, it is considered a reliable biomarker of brain tumour proliferation [59].

Owing to its low physiological uptake in normal brain tissue and the above-mentioned uptake mechanism, [^11^C]MET has been proved to be a sensitive radiopharmaceutical in tumour diagnosis, being able to identify even low-grade glioma often not detectable by [^18^F]FDG. For example, it has been demonstrated that [^11^C]MET PET is useful to differentiate tumorous from non-tumorous lesions in children and young adults with high diagnostic performance, particularly when routine structural imaging is equivocal [60]. In addition, [^11^C]MET PET may disclose, even in the context of non-enhancing and low grade gliomas, areas with more aggressive biological behaviour, representing preferred target for biopsy [56, 61, 62]. Furthermore, a recent study in patients with paediatric high-grade gliomas suggested that [^11^C]MET PET is superior to contrast-enhanced MRI in terms of diagnosing tumour recurrence [63].

Finally, it is important to underline that [^11^C]MET PET plays an important prognostic role in paediatric high-grade gliomas, which show diverging radiological features when compared with their adult counterparts [64]**.** Indeed, paediatric high-grade glioma may not show the classical imaging features of tumour aggressiveness (i.e. intense or inhomogeneous contrast enhancement on MRI), and intense [^11^C]MET uptake can provide important information about the presence of regions with intense tumour proliferation and neo-angiogenesis, which might be at high risk of disease progression [65]**.** This ability of [^11^C]MET PET to identify tumours that are at higher risk of relapse was confirmed in a subgroup of paediatric midline gliomas, such as the DIPG, whose grade and aggressive behaviour are particularly difficult to predict by analysing only MRI features [66].

#### [^18^F]FET

In the last two decades, novel fluorinated amino acid PET radiopharmaceuticals have emerged as effective alternative radiolabelled compounds to [^11^C]MET for studying paediatric gliomas. [^18^F]FET is a well-established amino acid for PET imaging in adults [28, 29] and offers clear logistic advantages over [^11^C]MET for clinical practice. The uptake mechanism is, like the other amino acid PET radiopharmaceuticals, governed by transport via specific amino acid carrier systems [67]. However, although [^18^F]FET is an analogue of L-tyrosine, which is a catecholamine precursor, it is not incorporated into any metabolic pathway [67]. Indeed, considering the similar uptake mechanism, comparative studies in adult brain tumours have shown that [^11^C]MET and [^18^F]FET may provide comparable results [68]. However, the advantage of [^18^F]FET over [^11^C]MET is the longer half-life (110 min vs 20 min) allowing radiopharmaceutical supply to facilities without in-house production, more patient evaluations per production, and the possibility to evaluate the time–activity curve (TAC) whose pattern may provide valuable additional diagnostic information.

Similar to [^11^C]MET, [^18^F]FET allows also in paediatric patients with non-enhancing and/or low-grade glioma to identify areas with more aggressive biological behaviour, representing preferred target for biopsy [69, 70].

[^18^F]FET static parameters such as tumour-to-background-ratio (*TBR*_max_ = *SUV*_max of the tumour_ / *SUV*_mean normal brain tissue_) are useful for interpretation. The addition of [^18^F]FET PET to MRI and combined reading increased accuracy of tumour detection in untreated and treated lesions [71]when using a threshold of *TBR* > 1.6.

The dynamic parameters can assist in the discrimination between low-grade and high-grade paediatric glioma at primary diagnosis [71] and in the differentiation between treatment related changes and glioma relapse [72, 73]. TAC with an early peak (≤ 20 min), followed by a constant descent, are associated with recurrence.

On the contrary, TACs with a constantly increasing pattern or a late peak at greater than 20–40 min after injection are often associated with non-neoplastic lesion (e.g. inflammation/infection, demyelinating or ischemic lesions) [72]. This approach may provide the best accuracy rate (about 82%) with a very high specificity (about 90%) [72]. Static [^18^F]FET images can also be of great help to distinguish post treatment changes from persistence of disease early after surgery, showing a higher specificity when compared with MRI, especially when the activity of the lesion presents a *TBR*_max_ > 2 [74].

Overall, the addition of [^18^F]FET PET to conventional imaging modalities is able to prompt modifications in the clinical management in about 8% of patients and to integrate relevant clinical information in 26%. The additional value provided by [^18^F]FET PET is related to its high specificity, especially in the case of suspected disease relapse [71].

#### [^18^F]DOPA

Intracellular uptake of [^18^F]DOPA is predominantly determinated by active transport systems carrying this radiopharmaceutical into the tumour. However, the uptake mechanisms of [^18^F]DOPA in brain tumours are not fully understood. Indeed, a significant increase in L-type amino acid transporter 1 (LAT1) expression has been determined in glioma; such increase has been found to correlate with gliomas [^18^F]DOPA uptake both in vitro and in vivo [75]. However, [^18^F]DOPA glioma uptake is not entirely dependent on LAT 1 expression [76]. Indeed, it has been reported that also the glutamine transporter sodium-coupled neutral amino-acid transporter (SNAT) 1 (Slc38a1) can drive [^18^F]DOPA uptake of cell lines of pHGGs [77]. Indeed, glutamine integration in high-grade gliomas is widely recognized [78].

Of note, H3K27M-mutant paediatric and adult diffuse midline gliomas are able to express dopamine receptor (DR) D2 [79] which have been showed to be functionally coupled with G protein that, in turn, promotes tumour growth. Moreover, an autocrine signalling process based on dopamine production has been demonstrated in glioblastoma cells [80]. Indeed, DOPA is a precursor in the dopamine synthesis and its uptake could represent a specific biomarker of cells that synthesize and secrete dopamine.

Studies demonstrated that [^18^F]DOPA PET results are associated to WHO tumour grade and outcome in paediatric infiltrative gliomas; moreover, they can be used for biopsy planning and treatment response assessment (e.g. discrimination between non-enhancing disease progression and treatment-related changes) [81, 82].

In differentiating paediatric non-neoplastic lesions from supratentorial infiltrative gliomas, MRSI demonstrated higher sensitivity and accuracy than [^18^F]DOPA PET [82]. The lower sensitivity of [^18^F]DOPA PET can be ascribed to the high prevalence of negative results in paediatric low-grade diffuse astrocytomas, which are considered separate and less aggressive tumours when compared with their adult counterpart [82].

In the setting of multimodal imaging of paediatric patients with diffuse astrocytic tumours, [^18^F]DOPA uptake correlated better with outcome and was an independent predictor of progression free survival when compared with MRI-derived diffusion weighted imaging (DWI) and arterial spin labelling (ASL) perfusion imaging [83]. In paediatric patients with H3K27M-mutant and wild-type DMG, [^18^F]DOPA PET was the only technique which was able to differentiate between H3K27M-mutant and wild-type lesions, when compared with MRI-derived DWI, MRSI, and ASL. This highlights its potential role to determine the H3K27M mutation status non-invasively [27].

In newly diagnosed DIPGs, [^18^F]DOPA PET imaging provided useful non-invasive information for evaluating their metabolism when compared with conventional MRI. The [^18^F]DOPA PET static parameter of target to contralateral striatum ratio (TSR) was an independent predictor of overall survival and [^18^F]DOPA uptake extent corresponded to more aggressive tumour component, which may be less sensitive to first-line treatment with chemotherapy/radiotherapy [77].

In this context, fused[^18^F]DOPA PET/MRI has shown to have a relevant role in patients treated with antiangiogenic agents in which it can early identify tumour “pseudoresponse” and non-enhancing tumour progression [84]. A more recent pilot study suggested that [^18^F]DOPA PET is of value to assess early response to bevacizumab in children with glioma relapse [85].

The main potential limitation of [^18^F]DOPA, compared with other amino acid radiopharmaceuticals, is its specific uptake in the striatum that could impair the ability to evaluate basal ganglia tumoural infiltration. However, in paediatric patients with glioma, [^18^F]DOPA PET has been reported to properly identify striatal involvement for lesions with a *TSR* > 1. On the other hand, [^18^F]DOPA striatal uptake implies the possibility to stratify tumour uptake further by enabling the calculation of two TBR indices (tumour/parenchyma and tumour/striatum) [86].

## Radiopharmaceuticals

All radiopharmaceuticals considered in these guidelines ([^18^F]FDG, [^11^C]MET, [^18^F]FET, and [^18^F]DOPA) must be produced by a qualified and certified company or by qualified personnel in a well-equipped radiochemistry/radiopharmacy unit. All the methods applied should satisfy regulatory requirements [28, 45, 87]. Quality controls of PET radiopharmaceuticals are usually performed by the producer.

According to the 2016 Update of the North American Consensus Guidelines for Paediatric Administered Radiopharmaceutical Activities and the 2016 EANM paediatric Dosage Card [88, 89], the administered activity should be the lowest possible but has to guarantee diagnostic image quality.

The radiation exposure depends on the injected activity and on the CT component. As reported above, this component should be optimized for adequate attenuation correction but also to avoid futile irradiation. Indeed, in the particular setting of paediatric gliomas, no significant information can be obtained from the CT component. Thus, an adequate PET/MRI co-registration is crucial and can be easily implemented by using various dedicated software tools.

Although for [^18^F]FDG an established administered activity has been widely accepted [89], for amino acid PET radiopharmaceuticals, the proposed administered activity is not yet standardized. However, by considering the published studies, the appropriate activity to administer can be determined according to Table 1. Currently, no minimum administered activity has been determined for brain [^18^F]FDG or amino acid imaging.

**Table 1:** Examples of injected activity and effective dose for each tracer

Age	Weight* (kg)	D/A (mGy/MBq) Urinary Bladder Wall**				E/A (mSv/MBq)(ICRP, 2015. Radiation dose to patients from radiopharmaceuticals: a compendium of current information related to frequently used substances. ICRP Publication 128. Ann. ICRP 44(2S). n.d.)				A (MBq)				E(mSv)			
		[^18^F]FDG	[^18^F]FET	[^18^F]DOPA	[^11^C]MET	[^18^F]FDG	[^18^F]FET	[^18^F]DOPA	[^11^C]MET	[^18^F]FDG [52]	[^18^F]FET [74]	[^18^F]DOPA [27]	[^11^C]MET [52, 90]	[^18^F]FDG	[^18^F]FET	[^18^F]DOPA	[^11^C]MET
1 y	9.7	4.7E-01	3.0E-01	1.0E + 00	5.1E-01	9.5E-02	8.2E-02	1.0E-01	4.7E-02	2.1–3.0 MBq/kg	3.0 MBq/kg	4 MBq/kg	5.5 MBq/kg	1.9–2.8	2.4	3.9	2.5
5 y	19.8	3.4E-01	2.2E-01	7.8E-01	2.8E-01	5.6E-02	4.7E-02	7.0E-02	2.5E-02					2.3–3.3	2.8	5.5	2.7
10 y	33.2	2.5E-01	1.6E-01	5.7E-01	1.8E-01	3.7E-02	3.1E-02	4.9E-02	1.6E-02					2.6–3.7	3.1	6.5	2.9
15 y	56.8	1.6E-01	1.1E-01	3.8E-01	1.2E-01	2.4E-02	2.1E-02	3.2E-02	1.1E-02					2.9–4.1	3.6	7.3	3.4

^*^(ICRP, 2015. Radiation dose to patients from radiopharmaceuticals: a compendium of current information related to frequently used substances. ICRP Publication 128. Ann. ICRP 44(2S). n.d.)

^**^Critical organ

*D/A* absorbed dose per activity administered, *E/A* effective dose per activity administered

## PET acquisition protocol

The patients should be positioned in the gantry with the head in a dedicated headrest and the arms along the body; the position should be maintained for the duration of the examination. Then, it should be accurately checked that the entire brain is located within the field of view. Once this is done, using flexible head restraints is very helpful to improve the stability of the head. After the rapid CT acquisition, the presence of parents or caregivers during the PET acquisition may be helpful to reassure the children and avoid head movements. Finally, if available, a monitor system for the patients’ condition and movements should be implemented during the entire acquisition.

For PET/CT, a single field of view acquisition should be performed with a low-dose CT component executed only for attenuation correction. In this setting, the CT parameters should be chosen to provide the lowest dose possible.

For PET/MRI, the acquisition protocol depends on the type of technique applied, which can be sequential or simultaneous [91–93]. In sequential imaging, the PET and the MRI scanners are positioned in-line with the patient tabletop moving between the two gantries [91]. Following the advent of semiconductor detectors, including avalanche photodiode or silicon photomultipliers, replacing former photomultiplier tubes, integrated PET/MRI scanners have been implemented which allow for a simultaneous acquisition of both components. The simultaneous PET/MR imaging is to be preferred for the paediatric population, given the significantly reduced scanning time and reduced need for patient sedation or anaesthesia [91]. Attenuation correction in PET/MRI systems are based on MRI imaging. Various correction strategies have been implemented and some may lead to systematic errors [94]. Care should be taken to identify artefacts in the attenuation correction, particularly in bone and lung [95]. Indeed, as the image quality of newer stand-alone PET/CT and MRI have improved compared to present generation PET/MRI, the scanner choice should be determined by weighting the expected image quality obtained with the discomfort of repeated examinations.

For PET/MRI sequences, axial fluid attenuation inversion recovery (FLAIR), T2-weighted and T1-weighted images, coronal FLAIR and T2-weighted images, and contrast-enhanced (gadolinium chelate, 0.1 mmol/kg) axial, coronal, and sagittal T1-weighted images are performed in addition to those for attenuation correction with a typical total acquisition time of 20–40 min depending on sequence composition and radiopharmaceutical [91, 96–98].

PET acquisition protocol should be adapted to the radiopharmaceutical used. These are briefly summarized in Table 2:

**Table 2:** Acquisition protocols for [^18^F]FDG and amino acid PET tracers

Radiopharmaceuticals	Time interval between radiotracer injection and image acquisition	Type of acquisition	Acquisition time
[^18^F]FDG	45–60 min	Static	10 min
[^11^C]MET	10 min	Static	10–20 min
[^18^F]FET	20 min (static), immediately after injection (dynamic)	Static and dynamic	10–20 min (static), 40–50 min (dynamic)*
[^18^F]DOPA	10–20 min	static	20 min

^*^Dynamic PET acquisition should be acquired in list mode and the properly reframed (short frame first and then longer ones) to provide precise information about the initial and late uptake phases which are essential to obtain the curve slope

## Image reconstruction

There are no reconstruction procedures specific to paediatric patients. How to implement image reconstruction is defined in previous guidelines [28].

## Image interpretation

Prior to image interpretation, an adequate quality check of the images (e.g. for movement artefacts) including factors that may affect the semiquantification (i.e. SUV) should be performed (e.g. activity, weight, and height should be correctly reported).

In the case of PET/CT, co-registration with recent MRI images should be always performed (i.e. at least with T1 sequence with contrast medium and T2/FLAIR sequence). Automatic or semiautomatic co-registration is strongly recommended. This allows precise evaluation even of faint uptake areas and easy identification of any brain lesion that needs metabolic characterization without having to adjust the PET display setting from default.

Visual analysis is the “corner stone” for correct interpretation of PET findings. Although SUV should be calculated when needed, its absolute value bears, per se, limited clinical relevance (see below). As the first step, for interpreting static PET images, the brain lesions should be identified, and their uptake should be assessed qualitatively. In the case of a non-negligible uptake (i.e. more intense than white matter), a semi-quantification should be performed by using the contralateral normal brain tissue activity as a reference (i.e. VOI including white and grey matter, measured at the level of the *centrum semiovale*) [31]. The target to background ratios (as *TBR*_mean_ and *TBR*_max_) should be calculated. Both work for [^18^F]FDG, [^11^C]MET, and [^18^F]FET, but for [^18^F]DOPA, the TSR should always be used in addition to TBRs. In the case of anaesthesia or sedation, TBRs can be affected; for example, a reduction in cortical [^18^F]FDG uptake can be expected especially in the parietal and occipital regions [99].

## Interpretation of static amino acid PET data

1. In the case of suspicious brain lesion on MRI, any detectable uptake (higher than normal background) may guide a selective biopsy. In this setting, no TBR threshold has been proposed.
2. At the time of first diagnosis, an important distinction should be made between suspected gliomas with diffuse infiltrative pattern vs. gliomas with a more circumscribed pattern of growth on MRI (Table 3).

**Table 3:** The 2021 World Health Organization classification of gliomas of the central nervous system [9] combining histopathological and molecular features into diagnoses according to the two different growing patterns which may influence PET/CT interpretation

Diffuse infiltrative pattern
*Paediatric-type diffuse low-grade gliomas*
Diffuse Astrocytoma, MYB- or MYBL1-altered
Angiocentric glioma
Polymorphous low-grade neuroepithelial tumour of the young
Diffuse low-grade glioma, MAPK pathway-altered
*Paediatric-type diffuse high-grade gliomas*
Diffuse midline glioma, H3 K27-altered
Diffuse hemispheric glioma, H3 G34-mutant
Diffuse pediatric-type high-grade glioma, H3 wild type, and IDH wild type
Circumscribed growth pattern
*Circumscribed astrocytic gliomas*
Pilocytic astrocytoma
High-grade astrocytoma with piloid features
Pleomorphic xanthoastrocytoma
Subependymal giant cell astrocytoma
Chordoid glioma
Astroblastoma, *MN1*-altered
*Glioneuronal and neuronal tumours*
Ganglioglioma
Desmoplastic infantile ganglioglioma/desmoplastic infantile astrocytoma
Dysembryoplastic neuroepithelial tumour
Diffuse glioneuronal tumour with oligodendroglioma-like features and nuclear clusters
Papillary glioneuronal tumour
Diffuse leptomeningeal glioneuronal tumour
Rosette-forming glioneuronal tumour
Myxoid glioneuronal tumour
Multinodular and vacuolating neuronal tumour
Gangliocytoma
Dysplastic cerebellar gangliocytoma (Lhermitte-Duclos disease) Central neurocytoma
Extraventricular neurocytoma
Cerebellar liponeurocytoma

A) In the case of a brain lesion with diffuse infiltrative pattern without contrast enhancement on MRI, the absence of uptake or faint uptake is mainly associated with low-grade gliomas. High-grade gliomas may be excluded, although midline gliomas may not show significant uptake. This poorly avid uptake pattern may also exclude oligodendrogliomas which are characterized by intense uptake in adult patients regardless of their histopathological grading and are exceptionally rare in the paediatric population.
  On the other hand, when intense uptake is observed, a high-grade lesion should be suspected. These lesions may also be present with contrast enhancement on MRI. The degree of uptake intensity cannot provide a distinction between WHO grades 3 and 4. However, distinguishing between these grades has limited clinical relevance.
B) In the case of brain lesions with a more circumscribed growth pattern and lack of contrast enhancement on MRI, absence of uptake or faint uptake is more likely associated with non-neoplastic lesions, although some low-grade gliomas (e.g. dysembryoplastic neuroepithelial tumour) cannot be excluded.
  In this circumscribed growth pattern, intense radiopharmaceutical uptake and contrast enhancement on MRI may often be associated with low-grade gliomas. Indeed, low-grade gliomas, such as pilocytic astrocytomas or gangliogliomas, are often characterized by intense radiopharmaceutical uptake. This is caused by the fact that these low-grade lesions may have significant neovascularization that presents a fenestrated epithelium, thus favouring an abundant radiopharmaceutical uptake [100, 101].

3) At the time of suspected relapse, intense uptake is highly specific for recurrent or residual tumour and may exclude the hypothesis of treatment-related changes (i.e. pseudo-progression or radio-necrosis).
4) In the case of ascertained gliomas with infiltrative growth pattern, the uptake intensity is often associated with prognosis, making the uptake intensity a very independent risk factor.

The most common uptake thresholds suggested in the literature to define a positive amino acid PET scan are summarized in Table 4.

**Table 4:** Commonly used TBR thresholds for amino acid PET in paediatric gliomas

Clinical Indication	Amino acid Radiopharmaceutical	Method	Threshold	Reference
Differentiation between neoplastic a non-neoplastic brain lesion	[^18^F]FET	TBRmax	1.6	[71]
	[^11^C]MET	TBR max	1–1.5	[56, 58, 102]
	[^18^F]DOPA	TBRmax	1	[81, 82]
Tumour Grading	[^18^F]FET	TAC curve shape	N.A	[72]
	[^11^C]MET	N.R	N.R	
	[^18^F]DOPA	TSRmax	1	[81, 82]
Prognostic value	[^18^F]FET	TBR max and TAC curve shape	1.6	[71, 72]
	[^11^C]MET	SUVmax	3	[65]
	[^18^F]DOPA	TSRmax	1	[81, 82]
Tumour Relapse	[^18^F]FET	TBRmax	1.6	[71]
	[^11^C]MET	SUVmax	3	[65]
	[^18^F]DOPA	Not available	Not available	Not available

N.R. = not reported, N.A. = not applicable

N.R. = not reported, N.A. = not applicable

## Interpretation of dynamic [^18^F]FET PET data

Only for [^18^F]FET, there is literature available, reporting the diagnostic role of dynamic parameters in paediatric patients [72, 74]. Three different patterns of time-activity curves are reported:

First, a rapid intake phase with subsequent further accumulation without any decreasing phases over time. This pattern is typical for benign or low-grade gliomas or with treatment-related changes when a relapse is suspected.

The second and third patterns are characterized by a first intake phase followed by a plateau or by a rapid decline in activity, respectively. These two curve shapes are associated with high-grade tumour at the time of initial diagnosis and may be indicative of relapse at follow-up, when disease recurrence is suspected.

For [^18^F]DOPA, as reported in adults patients, dynamic acquisition and related imaging interpretation could potentially provide additional information to static images [103].

## Interpretation of static [^18^F]FDG data

### At initial diagnosis

As for amino acid PET radiopharmaceuticals, increased [^18^F]FDG uptake by gliomas with infiltrative pattern is correlated with higher-grade and worse prognosis [104]. Accordingly, low-grade gliomas typically have [^18^F]FDG uptake similar to or less than white matter uptake, whereas high-grade gliomas have usually an [^18^F]FDG uptake that is higher than that of the white matter. However, as reported for the amino acid PET radiopharmaceuticals, some low-grade gliomas with more circumscribed growth pattern (e.g. pilocytic astrocytomas and pleomorphic xanthoastrocytoma) are characterized by high [^18^F]FDG uptake [51].

### Suspected tumour recurrence

Even though intense [^18^F]FDG uptake in enhancing brain lesions close to the site of previous surgery is associated with disease relapse, the specificity of discriminating tumour recurrence from therapeutic related changes is suboptimal. Indeed, persistent high [^18^F]FDG uptake may be observed after radiation therapy. However, recurrent tumours may also have relatively low [^18^F]FDG uptake. In this setting, visual or semi-quantitative criteria, able to provide high diagnostic accuracy, are not available.

As reported in adult guideline, dynamic [^18^F]FDG PET/CT acquisition may be performed to evaluate the regional metabolic rate (MRglu) [28]. This parameter is used to correctly estimate the real glucose consumption of the tumour. Dissociation between SUV and MRglu following (and during) therapy is possible, probably because of a change in plasma clearance induced by drugs. In those cases, SUV will underestimate or overestimate response [105]. Therefore, dynamic scanning with simultaneous SUV and regional metabolic rate calculation can be considered when new treatments are explored. However, considering that limited data are available, this approach cannot be recommended in clinical practice at this time.

## Pitfalls

In the process of image interpretation, some pitfalls should always be considered before providing a structured report.

The most common brain pitfalls have already been reported in the previous Joint EANM/EANO/RANO practice guidelines/SNMMI procedure standards for imaging of gliomas using PET with radiolabelled amino acids and [^18^F]FDG [28] or in another dedicated study [106]. Previous recommendations also apply to paediatric patients, with some caveats [86, 107–109].

In detail, because an increased prevalence of developmental venous anomalies (DVAs) in the setting of paediatric patients with brain tumours has been reported [110], increased amino acid uptake associated with DVAs may lead to a false-positive interpretation of brain tumour metabolism, potentially resulting in a haemorrhagic complication if a biopsy involves a DVA [107, 111]. Increased amino acid uptake of diffusely infiltrating low-grade epileptogenic tumours and adjacent brain parenchyma should also be evaluated in the light of clinical and electroencephalographic findings related to seizure activity [86]. In the setting of paediatric brain tumour surveillance, focal increased striatal [^18^F]DOPA uptake should be carefully interpreted in the light of MRI findings and of pathological changes related to cortico-striatal connections [109].

## Documentation and reporting

How to provide a structured report and describe PET findings has already been discussed in previously published guidelines on paediatric [^18^F]FDG PET/CT for oncology [45] and on imaging guidelines of gliomas using PET with radiolabelled amino acids and [^18^F]FDG [28].

## Equipment specifications

Paediatric patients are imaged with the same PET/CT and PET/MRI scanners designed for adult patients. It is advisable to use state-of-the-art scanners to achieve high-resolution images keeping a balance between administered activity and duration of the acquisition.

CT acquisition should be optimized using dedicated paediatric protocols whenever possible, whereas MRI sequences should be adapted to the degree of patient cooperation [112].

Sedation/anaesthesia requirements need to be considered in the set-up of the exam, taking into account the additional instrumentation (pulse monitor, oximeter, etc.) and the necessity of continuous patient monitoring.

## Conclusion

Considering the increasing use of hybrid imaging with PET/CT and PET/MRI in paediatric glioma, new guidelines providing proper information about patient selection, preparation and precautions, useful radiopharmaceuticals, radiation exposure, acquisition protocol, and image interpretation are required.

This document reports the main applications of [^18^F]FDG and amino acid PET radiopharmaceuticals in paediatric gliomas but is unable to provide evidence-based answers to the questions about which are the most effective radiopharmaceuticals or which are the most important indications to drive clinical management. However, in recent years, a growing body of evidence has shown that fluorinated amino acid PET radiopharmaceuticals have several diagnostic and logistic advantages over [^18^F]FDG and [^11^C]MET, respectively.

Regarding image interpretation, co-registration with the MRI is pivotal for a proper analysis of PET data. In this context, a continuous and close interaction between the image specialists (i.e. nuclear medicine physician and neuroradiologists) is the main requisite to provide clinicians with more robust information to define tailored treatment strategies [113].

## Liability statement

This guideline summarizes the views of the EANM (Paediatric, Neuroimaging and Oncology and Theranostic Committees), SNMMI, SIOPE, and RAPNO. It reflects recommendations for which the EANM cannot be held responsible. The recommendations should be taken into context of good practice of nuclear medicine and do not substitute for national and international legal or regulatory provisions.

## Abbreviations

ALARA: As Low As Reasonably Achievable
[^11^C]MET: L-[methyl-^11^C]methionine
CNS: Central nervous system
CT: Computed tomography
DIPG: Diffuse intrinsic pontine gliomas
DMG: Diffuse midline gliomas
DR: Dopamine receptor
DVA: Developmental venous anomalies
DWI: Diffusion weighted imaging
EGFR: Epidermal growth factor receptor
[^18^F]DOPA: 3,4-Dihydroxy-6-[^18^F]fluoro-L-phenylalanine
[^18^F]FDG: 2-Deoxy-2-[^18^F]fluoro-D-glucose
[^18^F]FET: O-(2-[^18^F]fluoroethyl)-L-tyrosine
FLAIR: Fluid attenuation inversion recovery
IDH: Isocitrate dehydrogenase
LAT1: L-type amino acid transporter 1
MAPK: Mitogen-activated protein kinase
MRglu: Metabolic rate of glucose
MRI: Magnetic resonance imaging
MRSI: Magnetic resonance spectroscopic imaging
PET: Positron emission tomography
PET/CT: Positron emission tomography/computed tomography
pLGG: Paediatric low-grade gliomas
pHGG: Paediatric high-grade gliomas
SNAT: Sodium-coupled neutral amino-acid transporter
SUV: Standardized uptake value
TAC: Time–activity curve
TBR: Tumour-to-background-ratio
TSR: Target to contralateral striatum ratio
WHO: World Health Organization
VOI: Volume of interest
